# Associations between Dietary Intake and Cardiovascular Disease Risk in American Career Firefighters: An Observational Study

**DOI:** 10.3390/jfmk9030132

**Published:** 2024-07-27

**Authors:** Anna Peluso Simonson, Jacquelyn N. Zera, Paromita Banerjee, Brianne M. Baker

**Affiliations:** 1 Department of Exercise Science and Sports Leadership, John Carroll University, University Heights, OH 44122, USA; jzera@jcu.edu; 2 Department of Mathematics, Computer Science and Data Science, John Carroll University, University Heights, OH 44122, USA; pbanerjee@jcu.edu; 3 Department of Nutrition Sciences, Dominican University, River Forest, IL 60305, USA; bbaker@my.dom.edu

**Keywords:** diet, firefighters, first responders, cardiovascular disease, tactical athlete

## Abstract

Firefighters have demanding jobs, requiring high levels of fitness in stressful situations for operational readiness, yet many firefighters are at an increased risk of developing cardiovascular disease (CVD). Diet is an important factor contributing to the development of CVD. The purpose of this study was to describe the dietary intake of firefighters and examine the associations between dietary intake and the CVD risk. Forty-six male career firefighters (age = 41.2 ± 11.2 years; BMI = 29.2 ± 4.1 kg/m^2^; body fat = 21.7 ± 6.1%) enrolled in a fitness-focused wellness program completed a health survey and a fitness assessment. The survey responses and fitness assessment were used to calculate the Framingham CVD Risk Score. Data were analyzed using R, the residual assumptions were verified, and the alpha level was set at 0.05. The results revealed that firefighters consume a standard American diet, with the overconsumption of meat and underconsumption of fruits and vegetables. The average CVD risk approached the upper limit of low risk. The results also indicate that meat servings and preparation fat affect the CVD risk (R^2^ = 0.21, *p* = 0.006). The outcomes of this study can inform investigations aimed at improving operational readiness and reducing the CVD risk in firefighters by implementing a holistic approach combining dietary interventions with physical training.

## 1. Introduction

Firefighters have demanding jobs with unique physiological and psychological requirements. As is well described in the literature, the physiological demands of firefighters may include high-intensity work in physically challenging situations of thermal stress and/or heavy load carriage involving personal protective equipment and tools needed to perform specific job tasks [1,2]. Additionally, firefighters experience increased occupational stress and other negative health behaviors associated with shift work [3,4,5]. These unique demands may predispose firefighters to cardiovascular or cerebrovascular events [6], which is a leading cause of on-duty death [7]. In combination with the risks associated with the demands of firefighting, firefighters are also subject to an increased risk of developing cardiovascular disease (CVD) from factors linked with CVD in the general population, such as obesity, dyslipidemia, hypertension, diabetes, age, and smoking [7]. A combined report from the US Fire Administration and the Federal Emergency Management Agency (FEMA) indicates that, in 2022, of the 94 on-duty firefighter fatalities, 37 deaths (39.3%) were caused by stress or overexertion, with 33 deaths from heart attacks and four deaths from stroke. Two firefighters died from heart attacks during training exercises [8].

Dietary intake, although a well-established modifiable risk factor for CVD, is only just emerging as a focus of investigation in this population. Western dietary habits (highly processed foods, saturated fat, and sodium intake) such as the Standard American Diet (SAD Diet) have been linked to chronic diseases such as obesity and CVD [9,10,11]. Specifically, regular shift work, associated with career firefighters, has been shown to negatively impact dietary patterns [12,13,14]. Evidence suggests that variations in meal timing and frequency resulting from regular shift work are associated with obesity and metabolic disease [15] and that food consumption that is asynchronous with typical circadian rhythms (such as with shift work) can negatively impact health and increase disease risks [16,17,18]. Therefore, it has been shown that shift workers are more likely to consume energy-dense convenience foods [19] that are high in sodium, saturated fats, and added sugars, although this is not thoroughly studied.

Evidence suggests that interventions to improve dietary intake and patterns have a positive impact on CVD risk factors in the general population. The Mediterranean Diet and the Dietary Approaches to Stop Hypertension (DASH) Diet have been shown to have beneficial effects on systolic blood pressure, lipid profiles, and inflammatory markers [20,21,22] associated with CVD. Additionally, healthy behaviors tend to cluster together with the association between diet and physical activity (PA), which is well established in the general population [23,24]. Combining dietary interventions with PA could lead to a more holistic approach to improving CVD risk in firefighters. However, prior to the design of tailored interventions, the population must be fully understood. Therefore, the purpose of this study was to (1) describe the dietary intake of firefighters and (2) examine the associations between dietary intake and CVD risk. We hypothesized that the dietary intake of firefighters would reflect that of the Western Diet or SAD Diet and that the increased intake of meat would be associated with an increase in the CVD risk.

## 2. Materials and Methods

### 2.1. Experimental Design

This study was a descriptive analysis designed to examine the associations between dietary habits and CVD risk in local career firefighters participating in a PA-focused employee wellness program. Data were collected online and in the Exercise Science Laboratory in the Spring of 2021. Firefighters completed a series of health- and diet-related questionnaires prior to an annual fitness assessment, which consisted of resting measures to evaluate obesity and CVD risk (heart rate, blood pressure, height, weight, and waist and hip circumference measurements) and assessments of all major fitness components (cardiorespiratory fitness, muscular strength, muscular endurance, flexibility, and body composition). 

### 2.2. Participants

In total, 46 male career firefighters (described in Table 1) from two fire departments in a midwestern metropolitan city were enrolled in a fitness-focused employee wellness program through their respective departments. Prior to their arrival, subjects answered questions on a health screening questionnaire to assess their risks and completed their annual fitness assessment either on- or off-shift, depending upon the requirements of their respective departments. This study was approved by the Institutional Review Board at John Carroll University. Subjects were informed of the risks and benefits of participation prior to signing an approved consent document.

### 2.3. Food Frequency and Health Questionnaire

Prior to their annual physical fitness assessment, the firefighters completed an overall health survey that included 20 questions related to nutrition and dietary intake. The questions were adapted from the Food Frequency Questionnaire (FFQ) and were specifically worded to examine the quantity and quality of intake. Diet quantity question responses included specific numbers of servings, with questions including, but not limited to, (1) “*How many servings of meat, poultry, or eggs do you eat per day (3 oz. or 1 serving is about equivalent to a deck of playing cards)?*”; (2) “*How many servings of seafood do you eat per week (3 oz. or 1 serving is about equivalent to a checkbook)?*”; (3) “*How may servings of nuts/seeds/soy/beans/legumes do you eat per week (2 Tbsp of nuts/seeds or ½ cup of beans/legumes is equivalent to 1 serving)?*”; and (4) “*How many servings of vegetables do you eat per day (one serving = ½ cup of fresh, frozen, or canned vegetables; ¾ cup of vegetable juice; 1 cup of salad greens)?*”. Diet quality question responses were assessed with a binary response (“*Plant*” or “*Animal*”) or on a 5-point Likert scale with responses ranging from “*Always*” to “*Never*”. Examples of intake quality questions include (1) “*When you eat grains (such as bread, rice, pasta, cereal, etc.), how often would you say that half of the grains you eat are whole grains (such as 100% whole wheat bread, brown rice, whole wheat pasta, etc.)?*”; (2) “*How often do you use meat, poultry, or egg alternatives (this includes tofu, seitan, soy, etc.)?*”; and (3) “*When you eat (either on or off shift) does the oil used to prepare your food typically come from plants (vegetable oil, olive oil, avocado oil, etc.) or animals (butter, lard, beef tallow, etc.)?*”.

### 2.4. CVD Risk

The CVD risk was assessed using the General CVD Risk Prediction Calculator prepared by R.B. D’Agostino and M.J. Pencina [25] as part of the Framingham Heart Study. The Framingham Risk Score estimates the 10-year risk of developing clinical CVD. Additionally, the calculation of the Framingham CVD Risk Score also provides an estimate of the vascular age. Risk calculation begins at 30 years of age. Firefighters less than 30 years old were analyzed as if they were 30 years old. This CVD risk prediction was based on a model using nonlaboratory predictors that are frequently measured in primary care: age, BMI, systolic blood pressure, use of antihypertension medications, current smoking status, and diabetes status. 

For this investigation, use of antihypertension medications, current smoking status, and diabetes status were measured using a self-report questionnaire. The use of antihypertension medications was assessed by asking participants if they were “*currently being treated for hypertension*”. Smoking status was assessed by asking participants if they were a “*current smoker*”, which was defined as “*any cigarette use in the last 30 days*”. Diabetes status was assessed with the question “*Have you currently been diagnosed with Type I or Type II Diabetes?*”. Age was self-reported at the time of assessment, and the measurements of the BMI and systolic blood pressure are described below.

### 2.5. Anthropometrics and Physical Assessment

The body mass index (BMI) (kg/m^2^) was calculated using height and weight. Height was measured using a wall-mounted stadiometer to the closest quarter inch (Seca, Hamburg, Germany). Weight was measured wearing lightweight exercise clothing with shoes removed, using a Model 349KLX Health-o-Meter Professional digital scale (Pelstar, LLC./Health o meter Professional, McCook, IL, USA), to the nearest tenth of a pound.

Blood pressure was measured after the subject had rested for a minimum of 5 min in a chair with back support and their feet on the floor. Using auscultation with an appropriately sized blood pressure cuff with a sphygmomanometer and with a Littman stethoscope placed below the antecubital space and over the brachial artery of the right arm, one measurement of blood pressure was taken by one of two certified exercise physiologists, according to the American College of Sports Medicine guidelines [26].

### 2.6. Statistical Analysis

All data were analyzed using RStudio Version 1.3.1093 (R Core Team, Vienna, Austria, 2021). Residual assumptions and normality of errors were verified and the alpha level was set *a priori* at α = 0.05. Spearman correlations and a linear regression model were used to study the association between dietary intake and the CVD risk. There was severe multicollinearity present between the servings of meat per week and servings of meat per day; therefore, only the servings of meat per week were considered for the regression model. The CVD risk was considered to be the dependent variable and the servings of meat per week, servings of red meat per week, use of meat alternatives, use of animal or plant fats for cooking, servings of seafood per week, servings of plant-based protein per week, servings of dairy per day, use of dairy alternatives, servings of fruit per day, and servings of vegetables per day were the independent variables in the linear regression model. The final model was selected based on stepwise regression with the Akaike Information Criterion (AIC) for variable selection after accounting for multicollinearity in the model. The paired-samples Wilcoxon signed rank test was used to determine differences between age and vascular age.

## 3. Results

### 3.1. Statistical Analysis

Given the small sample of firefighters included in this investigation, a power analysis was conducted to strengthen the credibility of the results. Based on the final model, with three predictors and a sample of 42 firefighters with complete data sets, the effect size of the model was determined to be 0.27 with a computed power of 0.82. With the power of 0.82 and a 5% significance level, a small sample can reliably detect moderate to large effects.

### 3.2. Dietary Intake

**Figure 1:**
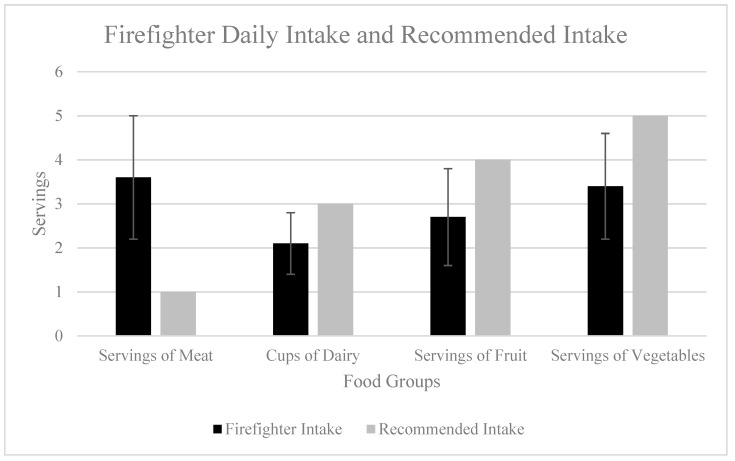
Comparisons between daily firefighter food group intake and American Heart Association (AHA) recommended intake.

**Figure 2:**
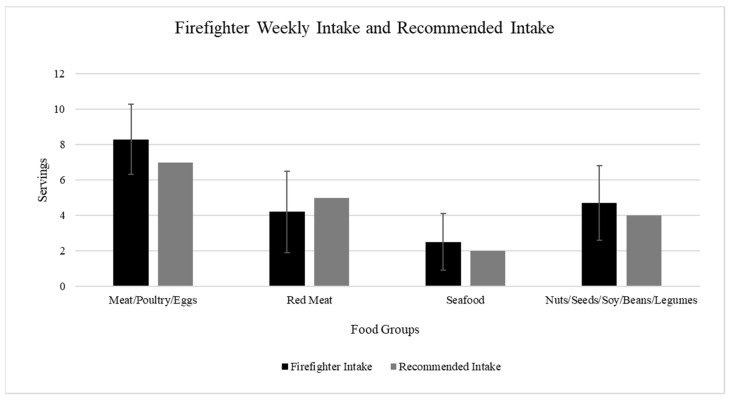
Comparison between weekly firefighter food group intake and AHA-recommended intake.

**Figure 3:**
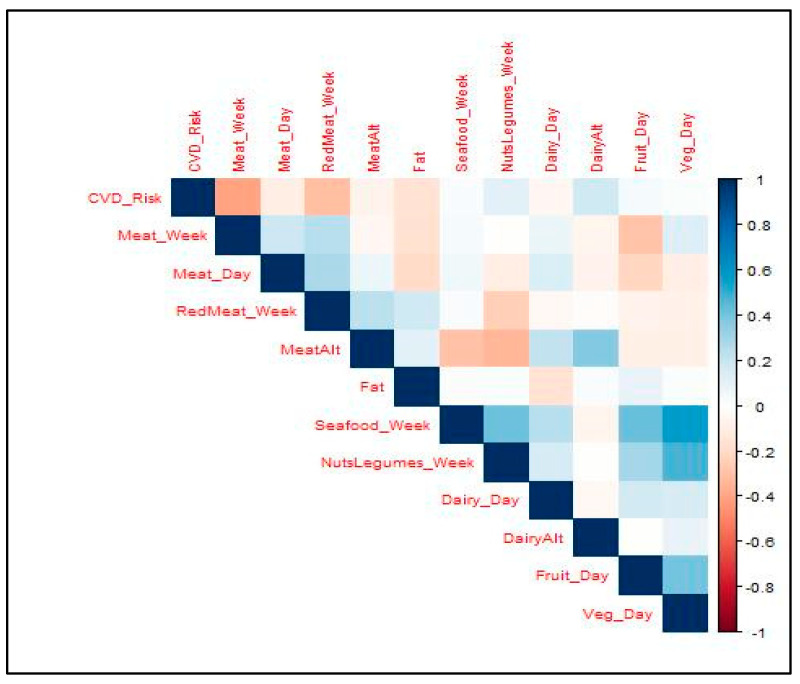
Correlations between dietary intake variables and Framingham Cardiovascular Disease (CVD) Risk.

The descriptive analysis of the dietary intake indicates that firefighters consume more than the American Heart Association’s (AHA) recommended amounts of meat per week, meat per day, seafood per week, and nuts/seeds/legumes per week, while consuming less than the recommended amounts of dairy per day, fruit per day, vegetables per day, and red meat per week [27] (Table 2, Figure 1 and Figure 2). The results of the one-sample t-tests indicate that all intakes, with the exception of nuts/seeds/legumes (*p* = 0.26), were significantly different from the recommended amounts (*p* = 0.00). Figure 3 exhibits the correlations between the dietary intake variables and Framingham CVD risk. The plot visually represents the correlation coefficients between multiple variables considered in the study. It shows that the highest correlation coefficients between the response variable of CVD risk were with the covariates of meat servings per week and servings of red meat per week. Given the recommendations to choose more plant-based options, consuming less red meat and more nuts/seeds/legumes per week would be a positive dietary behavior, while consuming more meat per week and per day would be considered a negative dietary behavior. 

Other healthy dietary behaviors clustered together. Servings of seafood per week was significantly and moderately correlated with servings of nuts/seeds/legumes per week (ρ = 0.39, *p* = 0.008), servings of fruit per day (ρ = 0.39, *p* = 0.006), and servings of vegetables per day (ρ = 0.44, *p* = 0.002). Servings of fruit per day and servings of vegetables per day were also significantly positively correlated with each other (ρ = 0.48, *p* = 0.001) (Figure 3). 

### 3.3. CVD Risk

The mean calculated CVD risk in this sample of career firefighters is 9.7 ± 6.5%, where 0–10% is considered a low risk, 10–19% is considered a moderate risk, and >20% is considered a high risk. While the mean calculated CVD risk was approaching the upper limit of a low risk, 33.3% of the participants were considered of moderate risk and 6.7% were considered of high risk. The results of the paired-samples Wilcoxon signed rank test indicated that the vascular age was significantly higher than the biological age (T = 6.00, *p* < 0.001) in this sample of career firefighters (Table 3).

### 3.4. Associations between Dietary Intake and Cardiovascular Disease Risk

The only significant correlations with the CVD risk are found for servings of meat per week (ρ = 0.39, *p* = 0.008) and servings of red meat per week (ρ = 0.319, *p* = 0.033) (Figure 3). The results of the stepwise linear regression indicate that a model using only the servings of meat per week and type of fat used in meal preparation significantly predicts the CVD risk. With an R^2^ = 0.21 and *p* = 0.006, approximately 21.42% of the variance in the CVD risk can be explained by the servings of meat per week and type of cooking fat (Table 4). Interestingly, the servings of meat per week was inversely related to the CVD risk, and, while the model was significant, the type of cooking fat alone was not a significant predictor of the CVD risk (β = −4.6, *p* = 0.111). The regression coefficient for the servings of meat per week was −1.4, indicating that with every increase in the servings of meat, poultry, or eggs per week, the risk of cardiovascular disease decreases by an average of 1.4 percentage points, with other variables held constant in the model. Note that the minimum number of servings of meat (including poultry and eggs) per week is five and the maximum is 10 for this group of firefighters. Moreover, the linear regression model is only interpretable within its range, i.e., we cannot infer from our model that consuming more than 10 meat servings per week will decrease the likelihood of developing cardiovascular disease as well. 

## 4. Discussion

This study takes a holistic approach to describing healthy lifestyle behaviors in this population. PA constitutes a major component of enhancing cardiorespiratory fitness (CRF), thus improving wellness and obesity management; however, PA alone is of limited benefit for weight loss and the reduction of the CVD risk. Dietary intake is an often undervalued yet critical factor in overall wellness, obesity, CVD prevention and treatment, and operational readiness. 

The dietary intake of this population of first responders aligns with the SAD diet. These dietary habits, consisting of the overconsumption of meat and minimal intake of fruit and vegetables, are independently associated with increased risks for obesity, CVD, type 2 diabetes, and certain types of cancer [11]. The reported intakes from this study are consistent with those of Lowden et al., who found that shift workers were more likely to eat convenience foods that were higher in fat and sodium and limited in fruit and vegetables [19]. Healthy dietary behaviors were shown to cluster together with other positive dietary behaviors. There were associations among the intake of nuts/seeds/legumes, seafood, fruits, and vegetables, suggesting that firefighters who consume fruits and vegetables tend to have a healthier dietary pattern across the food groups. In this investigation, dietary intake behaviors were self-reported, thereby introducing recall bias into the results. Therefore, an aim of future research should be to incorporate more accurate methods for the analysis of dietary intake, such as 3-day food recalls.

The cardiovascular disease risk in this population of career firefighters was at the higher end of the low risk category. These results differ from those of Gendron and colleagues, who found that a large percentage of Canadian firefighters (age 41.6 ± 10.4) were at a moderate to high risk of developing CVD [28]. The mean age of the current sample of firefighters was 41.2 ± 11.2 years, which is similar to the sample of Canadian firefighters. It is possible that the difference in the estimated risk is due to the method of calculation. The firefighters in the Gendron investigation completed an online lifestyle and CVD risk questionnaire, and the current study utilized a combination of questionnaires and measured outcomes to estimate the risk using the Framingham Risk Score. However, given that the CVD risk for firefighters under the age of 30 is inflated due to calculation limitations, it would appear that this population has an overall lower CVD risk than other similar populations. One additional factor that may account for this lower risk is that many of these firefighters had been participating in a fitness-focused wellness program for several years (between 2 and 7 years) and had already made behavioral changes to reduce their CVD risk, such as smoking cessation and increased physical activity, which could have affected their BMI, blood pressure, and diabetes status. Other studies have also investigated the CVD risk in firefighters [29,30]. However, these studies have counted the number of risk factors for participants but have not calculated a risk percentage, making it difficult to compare such studies.

The descriptive analyses suggest that local firefighters are classified as overweight, although the percentage of body fat is at the higher end of the healthy range for men in this age group (11.5–22.3%) [26]. The participants also had elevated levels of blood pressure, increasing their risk for CVD. The results of the current investigation align with the findings from the previous literature. A study by Fahs et al. found increased blood pressure and BMI in a population of young firefighters [31], while Garver et al. demonstrated elevated SBP and BMI in a different population of municipal firefighters [32]. Kales et al. discovered the same elevations in municipal and regional hazardous material response teams [33]. These results approach the results of Lemon and colleagues, who showed that a group of professional firefighters had body fat values that were similar to the sedentary population [34]. Poston et al. found higher rates of obesity in 677 firefighters than in the general population [35]. The findings from the current investigation, in conjunction with the previous research, indicate that increased rates of obesity (defined by BMI) and hypertension are extremely prevalent and consistent across types of first responders, particularly in different classifications of firefighters.

The results from the current study suggest that firefighters consume more than the recommended amount of meat. However, meat intake had a significant and negative correlation with the CVD risk and was the only dietary factor that was significant in predicting the CVD risk. Although the current recommendations suggest a reduction in meat intake and a focus on plant-based protein sources [27], the previous literature indicates that increased meat intake is not necessarily associated with an increased CVD risk. Several recent systematic reviews and meta-analyses have demonstrated that the evidence to suggest a link between meat intake and CVD is moderate at best [36,37,38,39]. However, there does seem to be a link between processed meat and CVD [36,37] that does not exist with unprocessed meat [36], particularly poultry or fish [36,39]. Additionally, nutritional recommendations for tactical athletes, particularly firefighters, indicate that these populations require a higher percentage of protein intake [40]. Future research investigating meat consumption and its association with the CVD risk should aim to separate the types of meat into red, processed, unprocessed, poultry, and fish to more fully understand the impact of meat intake on cardiovascular health.

Other studies have begun to examine the efficacy of lifestyle interventions in improving the CVD risk and overall health in first responders. Korre et al. presented preliminary data to suggest that adopting a Mediterranean diet has promising results in improving cardiovascular health in first responders [41], and other interventions focusing on the implementation of the Mediterranean Diet in first responders are underway [42]. While previous interventions have focused on one aspect of the CVD risk (i.e., PA or diet), Gill et al. demonstrated that a 1-year lifestyle program that utilized electronic and online tools and personalized diet and exercise plans improved participants’ body weight and CVD risk factors [43]. Due to the unique occupational demands of first responders, interventions aimed at decreasing the CVD risk should address both PA and dietary behaviors, while incorporating strategies that directly relate to the added difficulties of shiftwork and work-related stress. Evidence from Panda suggests that implementing time-restricted eating may attenuate the increased risk of metabolic disease resulting from the disrupted circadian rhythms associated with shift work [16]. Additionally, these investigations should utilize a tailored approach to lifestyle interventions by accounting for the effect of the baseline weight on the weight gain or loss resulting from increased job stress [3]. Investigations utilizing lifestyle interventions should also strive to implement the recommendations for shift workers indicated by Lowden et al., such as eating breakfast; maintaining a normal day- and night-time pattern of eating as much as possible; avoiding high-energy, less nutrient-dense foods and high-sugar items, etc. [19]. Given the link between dietary behaviors and the CVD risk, in addition to the unique occupational demands of first responders, future wellness interventions must include tailored strategies to address the CVD risk factors.

There are many strengths of this investigation, which include the focus on an important and understudied population, the use of objective laboratory-based measures for anthropometrics and blood pressure, and the use of standardized and validated questionnaires to create food frequency and prediction models for the CVD risk. However, there are limitations to the current study that should be highlighted and addressed in future investigations. The methodology of this study utilized an FFQ to gather dietary intake data. As with any self-report measurement, recall bias is certainly a threat to validity; however, there are very few measures of dietary intake that are both accurate and easily administered. Given the challenge of measuring dietary intake, previous research has indicated that FFQs are within the scope and design of this study for the measurement of a pattern of dietary intake (and components) over time, which requires only general memory [44]. Moreover, this study utilized a convenience sample of 46 firefighters, which is a small sample size. Given the unique nature and culture of this career field and the associated physiological and behavioral demands, firefighters are challenging to recruit and research. Hong et al. reported on the challenges and successes of recruiting firefighters for research, and their suggested strategies of collaborating with key stakeholders and expansion to multiple recruitment avenues were used to recruit departments into the wellness program [45]. However, a power analysis resulted in a statistical power of 0.82, indicating that the regression model used in this investigation could reliably detect moderate to large effects. This study was also conducted in American career firefighters with small minority representation. The results are limited in their generalizability to minority populations and other types of firefighters, such as those outside of the United States or volunteer, cadet/recruit, or wildland firefighters due to the homogeneity of the study population. These variables may have impacted the findings observed in this investigation and should be addressed in future research examining the association between dietary intake and the CVD risk. 

## 5. Conclusions

The results of this investigation indicate that local firefighters have dietary intakes that are similar to the Standard American diet: high in meat and red meat intake and low in fruit and vegetable intake. In addition to undesirable dietary patterns, the first responder participants also had hypertension and an elevated BMI. The combination of these factors places first responders at an increased risk for the development of CVD. Tactical strength and conditioning coaches, exercise physiologists, wellness experts, and health coaches must incorporate nutrition and behavioral strategies into wellness programming to minimize the negative health effects of shift work as part of a holistic program to reduce the risks of CVD and injury, improve performance, and increase operational readiness. Because of the positive impact that previous lifestyle interventions have had on CVD risk factors in similar populations, future investigations into the efficacy of lifestyle interventions to reduce the CVD risk in first responders are warranted. 

## Figures and Tables

**Table 1:** Participant demographic characteristics.

	Firefighters (Mean ± SD) (n = 46)
Age (years)	41.2 ± 11.2
Years of Service (years)	16.9 ± 10.2
BMI (kg/m^2^)	29.2 ± 4.1
Body Fat (%)	21.7 ± 6.1
Resting Blood Pressure	
Systolic (mmHg)	140.2 ± 14.1
Diastolic (mmHg)	82.5 ± 10.9

**Table 2:** Participants’ intake of food group servings and cooking fat.

Dietary Intake (n = 46)	Mean ± SD or Percentage (%)
Servings of Meat per Week	8.3 ± 2.0
Servings of Meat per Day	3.6 ± 1.4
Servings of Red Meat per Week	4.2 ± 2.3
Servings of Seafood per Week	2.5 ± 1.6
Servings of Nuts/Seeds/Legumes per Week	4.7 ± 2.1
Servings of Dairy per Day	2.1 ± 0.7
Servings of Fruit per Day	2.7 ± 1.1
Servings of Vegetables per Day	3.4 ± 1.2
Plant vs. Animal Cooking Fat	89.1% Animal

**Table 3:** Calculated participant Framingham CVD Risk Scores.

CVD Risk Components (n = 46)	Mean ± SD or Percentage (%)
CVD Risk (%)	9.7% ± 6.5%
Vascular Age (years)	49.7 ± 12.5
CVD Risk Category	
Low	60.0%
Moderate	33.3%
High	6.7%

**Table 4:** Associations between dietary intake and Framingham CVD Risk Scores.

Independent Variables	β	β_std_ ^1^	*p*-Value
Y-Intercept	26.6	5.3	<0.000
Servings of Meat per Week	−1.4	0.4	0.003
Plant vs. Animal Cooking Fat	−4.6	−1.6	0.111

^1^ β_std_ is the standard error of the estimated β.

## Data Availability

The raw data supporting the conclusions of this article will be made available by the authors on request.
